# Lipid droplets at the interface of plant defense and pathogen exploitation

**DOI:** 10.3389/fpls.2026.1911736

**Published:** 2026-08-21

**Authors:** Nathalie Arvy, Marguerite Batsale, Sara Shakir, Léna Jambou, Claire Bréhélin, Sylvie German-Retana

**Affiliations:** 1 Biologie du Fruit et Pathologie, UMR 1332, Equipe de Virologie, Institut National de Recherche pour l'Agriculture, l'Alimentation et l'Environnement (INRAe), University of Bordeaux, Bordeaux, France; 2 Laboratoire de Biogenèse Membranaire, UMR5200, Centre National de la Recherche Scientifique (CNRS), University of Bordeaux, Bordeaux, France

**Keywords:** defense, hijack, lipid droplets, plant pathogens, proteome, transcriptome, virus

## Abstract

Lipid droplets (LDs) are increasingly recognized as dynamic organelles involved in lipid metabolism, organelle communication, and stress responses. During plant-pathogen interactions, LDs undergo extensive remodeling, including changes in abundance, lipid composition, intracellular distribution, and associated proteins. Recent studies indicate that bacterial and fungal infections consistently promote LD accumulation and proteome reprogramming. This supports emerging roles for LDs in antimicrobial metabolism, defense signaling, membrane remodeling, and cellular homeostasis. However, these organelles may also provide metabolic resources that pathogens exploit to promote infection. Beyond bacterial and fungal pathogens, recent evidence suggests that plant viruses could also manipulate LD biology to facilitate replication compartment biogenesis, membrane remodeling, and potentially virus movement. In this review, we summarize current knowledge on LD dynamics during plant-pathogen interactions, compare LD responses across different pathogen lifestyles, and discuss the dual roles of LDs in plant immunity. Finally, we propose a conceptual framework in which LD functions may support host defense, be actively manipulated by pathogens, thereby providing a broader perspective on the diverse roles of LDs in determining disease outcomes.

## 1 Introduction

Cytosolic lipid droplets (LDs) are dynamic multifunctional organelles that contribute not only to energy storage but also to the trafficking of lipids, proteins, and signaling molecules essential for cellular function (Murphy et al., 2009; Murphy, 2012; Olzmann and Carvalho, 2019). LDs are widespread across eukaryotes and are also found in some prokaryotes. They consist of a neutral lipid core, mainly triacylglycerols (TAGs) and sterol esters (SEs), surrounded by a phospholipid monolayer coated with a diverse set of associated proteins (Bouchnak et al., 2023; Klemm and Carvalho, 2024). In eukaryotic cells, LDs originate at defined sites of the endoplasmic reticulum (ER) through the synthesis and accumulation of neutral lipids between ER membrane leaflets into lens-like structures (Walther et al., 2017; Pyc et al., 2021; Guzha et al., 2023). The budding into the cytosol is then guided by SEIPIN proteins which oligomerize to form a ring at the ER outer leaflet, regulating lipid and protein transfer to the nascent LDs (Klug et al., 2024). While only one SEIPIN protein is present in yeast and animal cells, multiple SEIPIN isoforms are encoded in plant genomes. The formation of LDs is considered to occur in plants through a process similar to that described in yeast and animal cells, but it also relies on plant-specific factors including lipid droplet-associated proteins (LDAPs), their partner lipid droplet-associated protein-interacting protein (LDIP), and the tethering vesicle-associated membrane protein-associated protein 27-1 (VAP27-1), which both contribute to LD biogenesis by interacting with SEIPINs (Greer et al., 2020; Pyc et al., 2021). Whether plant LDs eventually dissociate from the ER remains an open question.

In plants, LDs are best known in seeds for their role in storing oil, which provides the energy needed for seedling growth after germination. However, they are also described in vegetative tissues, where they accumulate in response to diverse abiotic stresses such as drought, heat stress, or nitrogen starvation (reviewed in Bouchnak et al., 2023; Zhao et al., 2025; Dabisch et al., 2026). In addition to cytoplasmic LDs, plants also contain plastid-localized plastoglobuli whose size and abundance vary markedly with environmental conditions (Bréhélin and Kessler, 2008; van Wijk and Kessler, 2017; Coulon et al., 2024; Liu et al., 2025). However, in this review, we have chosen to focus exclusively on cytoplasmic LDs.

LDs are increasingly recognized as polyvalent organelles contributing to lipid homeostasis by storing membrane lipid precursors, supporting cellular integrity by sequestering lipotoxic molecules, but also participating to lipid and protein trafficking between organelles (reviewed in Bouchnak et al., 2023; Farese and Walther, 2025). During seed germination, mobilization of oil stored in LDs is essential for early plant growth (Hamade et al., 2025). TAGs mobilization relies primarily on lipolysis and lipophagy (reviewed in D’Andrea, 2016; Bouchnak et al., 2023; Qin et al., 2023). In seeds, lipolysis involves the removal of LD-associated proteins, primarily oleosins (OLEs), that form a protective coat on the surface of LDs, preventing lipases from accessing their TAG substrates. The ubiquitinated OLEs are extracted from the LD surface by the PUX10–CDC48 machinery and undergo proteasome-dependent degradation (Deruyffelaere et al., 2018; Kretzschmar et al., 2018). TAG breakdown and fatty acid (FA) transfer to peroxisomes are mediated by proteins such as SUGAR-DEPENDENT PROTEIN 1 (SDP1), a TAG lipase anchored to the peroxisomal membrane (Eastmond, 2006; Kelly et al., 2013), and the peroxisomal ABC transporter PXA1, which imports FAs into the organelle for β-oxidation (Zolman et al., 2001; Footitt et al., 2002; Hayashi et al., 2002). LD-peroxisome membrane contact sites (MCSs) are thought to facilitate the direct transfer of FAs from LDs to peroxisomes, thereby tightly coupling lipolysis with peroxisomal β-oxidation (Thazar-Poulot et al., 2015). Beyond its role in carbon and energy metabolism, peroxisomal β-oxidation also contributes to plant immunity through its involvement in jasmonic acid (JA) biosynthesis, in concert with chloroplast metabolism (Wasternack and Hause, 2013). However, the fatty acids released from LDs are not direct precursors of JA. Rather, LD-derived FA catabolism and JA biosynthesis rely on partly overlapping peroxisomal β-oxidation machinery. Changes in LD mobilization or in peroxisomal metabolic activity could therefore indirectly influence the allocation and regulation of peroxisomal pathways involved in defense. In parallel, peroxisomes are major hubs of reactive oxygen species (ROS) metabolism, integrating lipid catabolism with redox homeostasis (del Río and López-Huertas, 2016). Whether pathogen-induced remodeling of LD-peroxisome interactions affects these interconnected metabolic and defense functions remains an important question for future studies. Besides canonical lipolysis, LD degradation can also occur through lipophagy, a process reported during pollen maturation and germination (Kurusu et al., 2014; Zhao et al., 2025), following dark-induced leaf starvation (Fan et al., 2019), and during *Arabidopsis thaliana* (*Arabidopsis*) seed germination through a CLO1–ATG8-dependent microlipophagy-like process (Miklaszewska et al., 2023).

LDs are physically and functionally integrated within intracellular organelle networks. They interact with multiple compartments, including the ER, peroxisomes, mitochondria, plasma membrane (PM), and plasmodesmatas (PDs) in plants (Paul et al., 2014; Veerabagu et al., 2021; Krawczyk et al., 2022). These interactions can occur at MCSs, which enable direct exchange of molecules without membrane fusion. LDs have also been proposed to transport proteins to PDs and contribute to PD structure and function (Paul et al., 2014). Using live-cell imaging, Omata et al. (2024) showed that *Arabidopsis* leaf LDs exhibit actin-dependent movement and identified LD-associated myosin-binding proteins (MYOBs), raising the possibility that LD trafficking is driven by myosin motors coordinated by MYOBs.

LD protein composition differs across plant tissues: OLEs and caleosins are particularly enriched in seed or pollen LDs, whereas leaf LDs are enriched in LDAPs (Gidda et al., 2016; Brocard et al., 2017). OLEs and LDAPs are thought to stabilize LD architecture by limiting coalescence (Gidda et al., 2016). Several plant proteomic studies identified hundreds of LD-associated proteins, highlighting the versatility of these organelles (Brocard et al., 2017; Omata et al., 2024; reviewed in Dabisch et al., 2026).

LDs are also induced during plant response to environmental stresses. Thermal stress, for example, triggers LD accumulation and perturbs membrane properties, requiring lipid remodeling to maintain membrane function (Mueller et al., 2017; Yang et al., 2024). LDs that accumulate in response to such stress, are proposed to sequester FAs derived from plastid membrane degradation induced by stress (Mueller et al., 2015; Coulon et al., 2024). LD mobilization is also tightly regulated in response to salinity as highlighted by Wang et al. (2025) during post-germination growth arrest in *Arabidopsis*, a rapid and reversible adaptive mechanism. The protein composition of LDs varies in response to heat, drought, and cold stresses (Gidda et al., 2016; Doner et al., 2021; Scholz et al., 2025), supporting the view that plants deploy stress-specific LD reprogramming.

**Figure 1:**
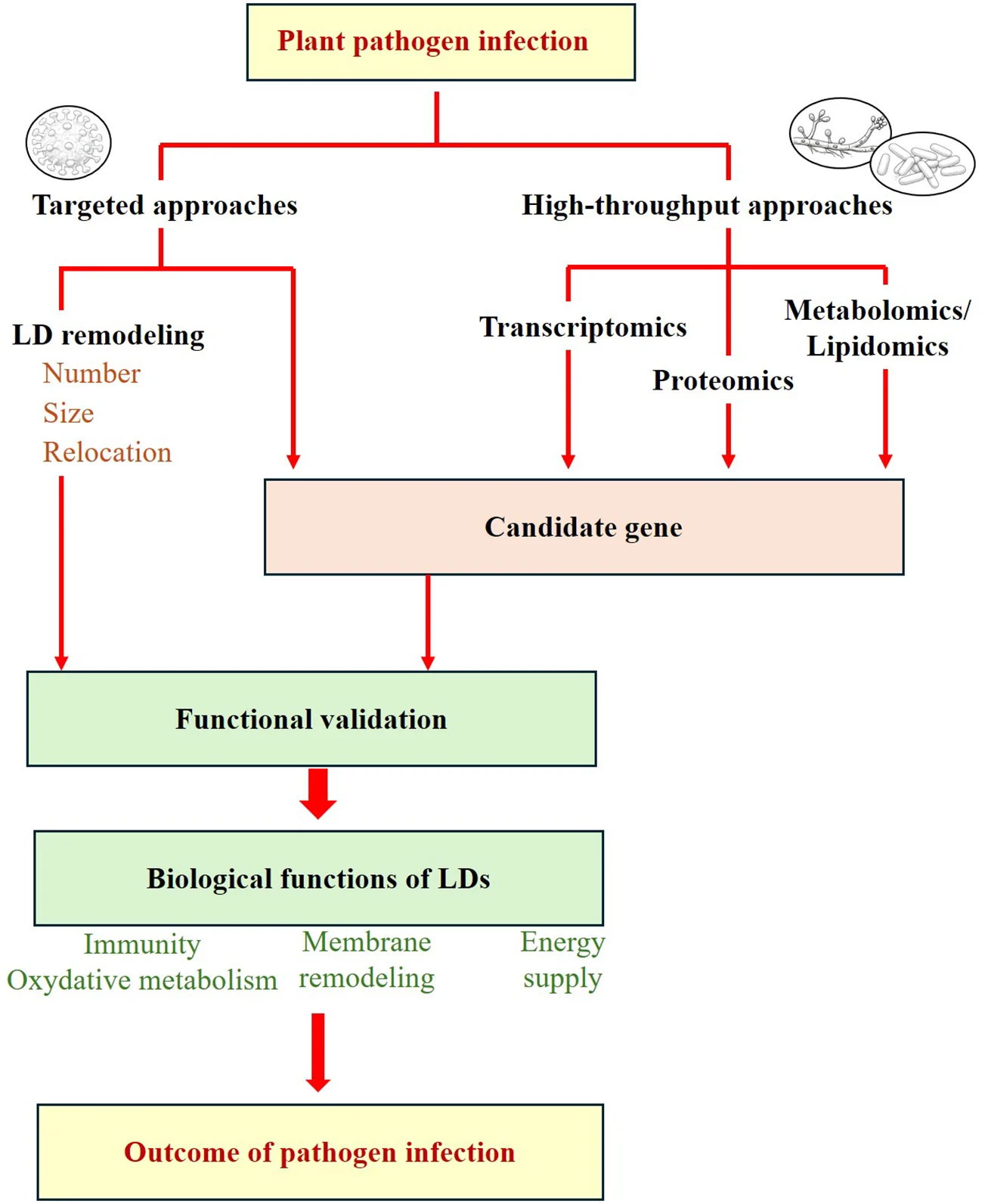
Integrated framework for investigating LD role during plant-pathogen interactions. High-throughput approaches, including transcriptomics, proteomics, metabolomics, and lipidomics, have provided extensive information on LD remodeling during bacterial and fungal infections, enabling the identification of candidate genes and pathways that further require functional validation. By contrast, our current understanding of LD functions during viral infection relies mainly on targeted approaches. This disparity in the level of available information is reflected in the organization of this review, with bacterial/fungal and viral interactions discussed in separate sections.

Accumulating evidence further indicates that LDs also participate in the plant response to biotic stresses. Indeed, TAG accumulation has been reported in leaves and roots in response to pathogens, especially fungi and bacteria (Schieferle et al., 2021; Li et al., 2023; Scholz et al., 2025). However, how LD dynamics, neutral lipid accumulation, and LD-associated proteins contribute to plant-pathogen interactions remains poorly described. In this review, we therefore focus on the involvement of LDs in the plant defense arsenal and examine how diverse pathogens may manipulate LD biology to their advantage, with an opening on plant viruses. As summarized in **Figure 1**, we present an integrated framework used so far to tackle the role of LDs during plant-pathogen interactions. We also draw on key insights from animal systems when relevant to address the central question of this review: what roles do LDs play in plant-pathogen interactions?

## 2 LDs in plant responses to bacterial and fungal pathogens: friends or foe?

### 2.1 Modulation of LD composition and dynamics during bacterial and fungal infections

Reports have highlighted a marked accumulation of TAGs together with an increase in LD number in infected tissues, pointing to a global reprogramming of neutral lipid metabolism. This metabolic shift is also associated with changes in LD protein composition, suggesting that LDs may participate in infection-specific metabolic and defense-related processes. In this section, we examine how bacterial and fungal infections reshape LD dynamics at multiple levels, from TAG accumulation to LD proteome remodeling, and discuss the underlying mechanisms as well as their functional implications during plant-pathogen interactions.

#### 2.1.1 Pathogen-induced TAG accumulation reveals a reprogramming of neutral lipid metabolism

Multiple studies have shown that pathogen infection significantly affects plant lipid metabolism, notably through the accumulation of TAGs. This response has been reported for phylogenetically distinct pathogens, including bacteria and fungi, suggesting that TAG accumulation represents a conserved metabolic signature of plant-pathogen interactions.

*Pseudomonas syringae* infection consistently leads to a strong increase in TAG levels in infected *Arabidopsis* leaves, as reported by several studies (Zoeller et al., 2012; Schieferle et al., 2021; Scholz et al., 2025). Lipidomic analyses indicate that the abundance of membrane lipids including galactolipids and phospholipids remains broadly unchanged, suggesting a TAG-specific response to *P. syringae* infection (Scholz et al., 2025). Consistent with these lipid analyses, confocal microscopy observations also revealed an increased accumulation of LDs following *P. syringae* infection (Fernández-Santos et al., 2020; Li et al., 2023). Comparable responses have been observed during fungal infections: infection by the powdery mildew *Golovinomyces orontii* results in a 3.5-fold elevation of TAG levels, consistent with an increase in both LD and plastoglobule abundance (Xue et al., 2025) and an increase in LD number was observed by confocal microscopy upon infection with the hemibiotrophic fungus *Colletotrichum higginsianum* (Shimada et al., 2014).

Further observations indicate that pathogen-induced TAG accumulation can occur either locally at infection sites or systemically in distal tissues, depending on the pathogen and its colonization strategy. In contrast to *P. syringae*-induced TAG accumulation, which remains largely confined to locally infected leaves, infection of *Arabidopsis* by the vascular fungal pathogen *Verticillium longisporum* triggers a systemic lipid response, with a 6-fold increase in TAG content observed both in infected roots and in distal leaves where no pathogen could be detected (Schieferle et al., 2021). This systemic lipid reprogramming suggests the involvement of long-distance signaling pathways that coordinate neutral lipid metabolism at the whole-plant level.

Although TAG accumulation emerges as a general feature of plant responses to infection, the metabolic pathways underlying this response remain poorly understood. Analysis of TAG molecular species provides useful, albeit indirect, insights into the origin of the FAs incorporated into TAGs. FAs incorporated into TAGs can derive from either membrane lipid remodeling or *de novo* synthesis. Consequently, TAGs enriched in polyunsaturated FAs are typically associated with membrane-derived acyl chains, whereas those containing more saturated and monounsaturated FAs are more likely to reflect the incorporation of acyl groups from *de novo* synthesized acyl pools (Li-Beisson et al., 2013). Across these different pathosystems, pathogen-induced TAG accumulation is consistently associated with a strong enrichment in highly unsaturated TAG species in leaves, as observed during *P. syringae*, *V. longisporum* and *G. orontii* infections of *Arabidopsis* (Schieferle et al., 2021; Scholz et al., 2025; Xue et al., 2025), which supports a membrane-derived origin of FAs incorporated into TAGs and points to active membrane lipid remodeling during infection. However, no decrease of the global abundance of major membrane lipids was observed upon *V. longisporum* and *P. syringae* infections in *Arabidopsis* leaves, suggesting a preserved overall membrane lipid homeostasis instead of an extensive membrane lipid breakdown in these pathosystems (Schieferle et al., 2021; Scholz et al., 2025). Contrasting results were observed during infection by *G. orontii* with a strong decrease in membrane lipid content including monogalactosyldiacylglycerol (MGDG), digalactosyldiacylglycerol (DGDG) and phosphatidylcholine (PC) (Xue et al., 2025). The variable impacts of different pathogens on host membrane lipids contents reflect distinct infection strategies and host-pathogen interactions regarding TAG metabolism in different pathosystems.

To further dissect the biosynthetic routes responsible for pathogen-induced TAG accumulation, Schieferle et al. (2021) analyzed TAG levels in *Arabidopsis* mutants defective in the canonical TAG biosynthetic enzymes diacylglycerol acyltransferase 1 (DGAT1) and phospholipid diacylglycerol acyltransferase 1 (PDAT1), following *V. longisporum* infection. DGAT1 catalyzes TAG synthesis using acyl-CoA substrates, whereas PDAT1 transfers acyl chains directly from phosphatidylcholine to diacylglycerol (Li-Beisson et al., 2013). Surprisingly, pathogen-induced TAG accumulation was still increased in both mutants, indicating that neither DGAT1 nor PDAT1 is strictly required for TAG accumulation in response to *V. longisporum*.

Altogether, although the enrichment of pathogen-induced TAGs in polyunsaturated FAs is consistent with a membrane-derived origin, direct evidence for the precise source of these acyl chains remains lacking. The apparent redundancy of TAG biosynthetic pathways and the dynamic exchange between lipid pools makes it difficult to elucidate the respective contributions of membrane remodeling and *de novo* synthesis. Addressing this question will ultimately require approaches capable of tracing lipid fluxes rather than steady-state lipid compositions. Another way to explore LD accumulation in infected tissues is through LD proteomics, which can identify protein partners linked to infection-associated mechanisms and metabolic pathways.

#### 2.1.2 Pathogen-induced remodeling of the lipid droplet proteome

In recent years, several studies have been dedicated to the characterization of LD proteomes as a functional entry point to decipher potential roles of non-seed LDs. As *Arabidopsis* leaves do not accumulate substantial amounts of LDs under standard growth conditions, most proteomic analysis were performed in response to plant aging and environmental changes, including leaf senescence, drought and heat stresses, and infection by *P. syringae* and *B. cinerea* (Brocard et al., 2017; Fernández-Santos et al., 2020; Doner et al., 2021; Scholz et al., 2025). In most cases, proteomic analyses were complemented by subcellular localization approaches to validate LD association for a subset of newly identified proteins. Recent studies have taken advantage of *Arabidopsis* mutants with high levels of LDs under standard growth conditions to facilitate the extraction of LDs for proteomic analyses: Omata et al. (2024) performed LD proteomic analyses under non-stress conditions using the *high sterol ester 1* (*hise1*) mutant with elevated SE levels (Shimada et al., 2019) whereas Scholz et al. (2025) used the *tgd1–1 sdp1–4* mutant with high TAG content (Fan et al., 2014) to compare LD proteomes between unstressed plants and plants exposed to heat stress or pathogen infection.

**Figure 2:**
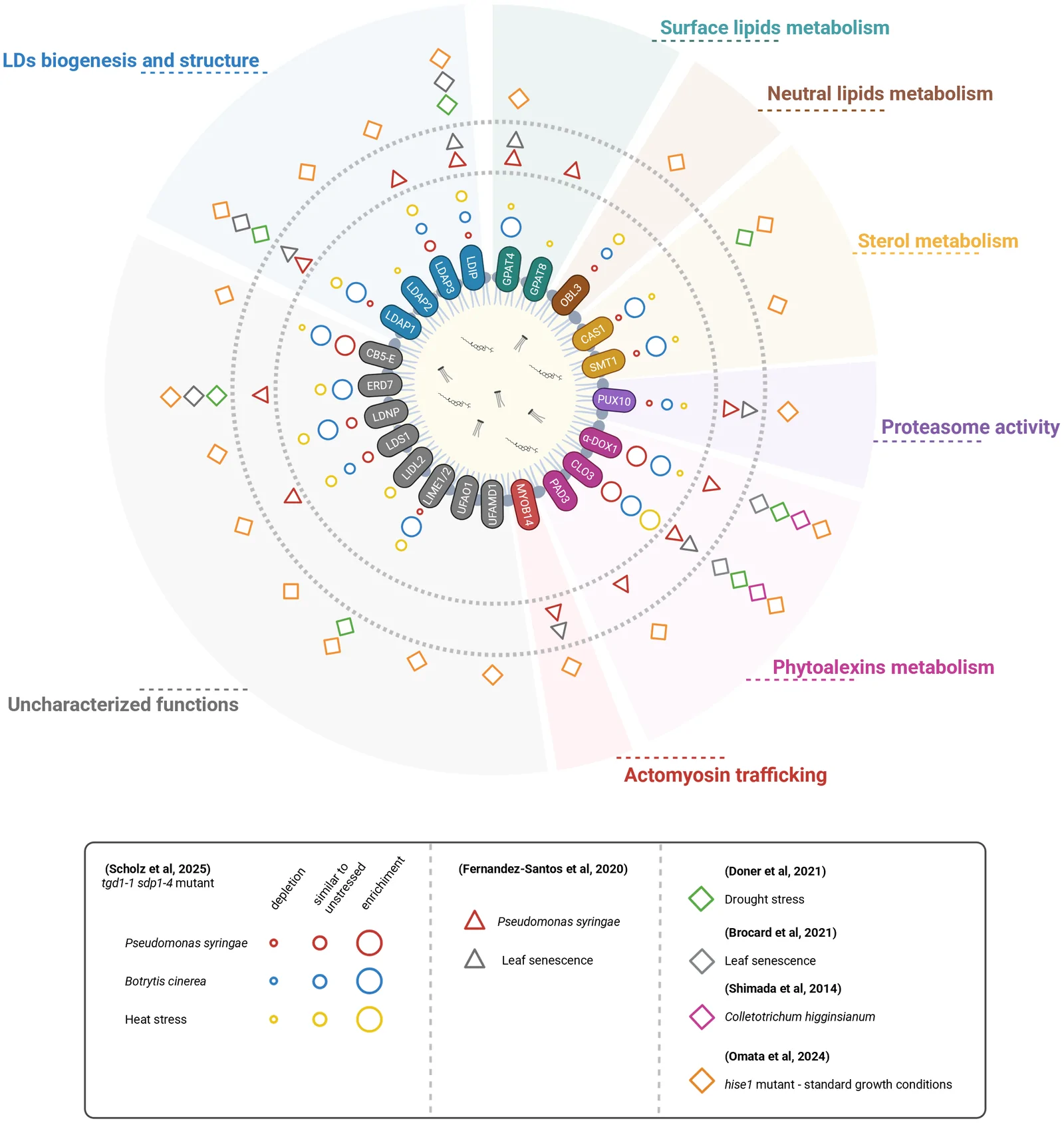
Remodeling of the leaf LD proteome in response to biotic and abiotic stresses. The concentric dashed circles correspond to independent proteomic studies. The inner circle corresponds to proteins identified in *Arabidopsis* leaf LDs from Scholz et al. (2025). The abundance of LD proteins under stressed condition (heat, *Botrytis cinerea* or *Pseudomonas syringae*) relative to unstressed control, is estimated by the ratio (stressed/unstressed control) and symbolized by a circle of different size: ratio < 0.5 (small circles), depleted; ratio 0.5–2 (medium circles), unchanged; ratio > 2 (large circles), enriched. This threshold was applied here for visualization purposes only and does not reflect a statistical significance cutoff established in the original study. In the middle circle, the data from Fernández-Santos et al. (2020) are symbolized by triangles that represent LD proteins detected in LD proteome of leaves infected with *Pseudomonas syringae* or senescent leaves of *Arabidopsis* plants. In the outermore circle, diamonds represent LD proteomes isolated under distinct experimental conditions, each corresponding to a separate study as specified in the legend panel below the figure. Proteins are arranged radially according to their functional category based on the annotated or predicted biological functions of LD-associated proteins. Because LD isolation protocols, quantification methods, and statistical thresholds vary across the studies compiled here, direct quantitative comparison of protein abundance across studies is not intended, the figure only provides a qualitative overview of LD-associated proteins identified across conditions. The meanings of the abbreviations are provided in the list of abbreviations. This Figure was created with BioRender.com.

Overall, proteomic analyses using *Arabidopsis* wild-type and mutant lines revealed a substantial overlap between the proteomes of LDs formed in response to biotic and abiotic stresses, along with consistent pathogen-specific differences (**Figure 2**).

The presence of some LDAPs and LDIP in every leaf LD proteome supports a fundamental role for these proteins in LD biogenesis, stability, and/or size regulation in response to both biotic and abiotic stresses (Fernández-Santos et al., 2020; Doner et al., 2021; Scholz et al., 2025): in contrast to LDAP1, which is present in every proteome (**Figure 2**), LDAP2 was only detected in high-LD-level mutants and seemed to be depleted upon heat stress (Omata et al., 2024; Scholz et al., 2025). Interestingly, LDAP3 accumulation in *Arabidopsis* wild-type plants was specifically observed in *P. syringae*-infected plants, whereas no significant detection was reported during senescence or drought stress (Brocard et al., 2017; Fernández-Santos et al., 2020; Doner et al., 2021). These observations suggest that LDAP3 may fulfill specialized functions in response to specific stresses, including pathogen infection.

In addition to structural components, LD proteomes display enzymes involved in different lipid metabolic pathways, suggesting that LDs are sites of active lipid synthesis and remodeling under environmental changes and pathogen infection. Among these, glycerol-3-phosphate acyltransferases GPAT4 and GPAT8 were identified. In *Arabidopsis*, GPAT4 and GPAT8 are bifunctional enzymes exhibiting both acyltransferase and phosphatase activities, leading to the production of monoacylglycerols (MAGs) that serve as precursors for cutin biosynthesis, a major component of surface lipids (Yang et al., 2012). In the comparative study led by Fernández-Santos et al. (2020), both GPAT4 and GPAT8 proteins were consistently detected in LD fractions isolated from wild-type *Arabidopsis P. syringae*-infected leaves, whereas only GPAT4 was significantly detected in LD fractions during senescence. When transiently expressed in *Nicotiana benthamiana*, *Arabidopsis* GPAT4 and GPAT8 were predominantly localized to the ER but partially relocalized to LDs following *P. syringae* infection. Together, these observations suggest a role for LDs in the metabolism of surface lipid precursors, with the association of cutin biosynthesis enzymes with LDs at least during *P. syringae* infection (Fernández-Santos et al., 2020).

The oil body lipase OBL3 was also identified in leaf LD proteomes in the *tgd1–1 sdp1–4* and *hise1* mutants (Omata et al., 2024; Scholz et al., 2025). OBL3 contains an α/β hydrolase domain characteristic of TAG lipases. The detection of TAG lipases in LD fractions supports the notion that LDs not only store neutral lipids but may also be dynamic organelles harboring enzymes directly involved in neutral lipid mobilization. OBL3 was detected in LD fractions under heat stress, infection with *P. syringae*, and infection with *B. cinerea* (Scholz et al., 2025).

Proteins involved in sterol metabolism, such as C-24 sterol methyl transferase 1 (SMT1) and cycloartenol synthase 1 (CAS1), were also detected in LDs of the *tgd1–1 sdp1–4* mutant under all tested stress conditions, but only infection with *B. cinerea* led to their enrichment in the LD proteome compared to the unstressed control (Scholz et al., 2025). Both enzymes participate in phytosterol biosynthesis, CAS1 catalyzes the cyclization of 2,3-oxidosqualene into cycloartenol and SMT1 mediates subsequent methylation steps in sterol formation (Corey et al., 1993; Husselstein et al., 1996). CAS1 was also identified in the LD proteome under drought stress (Doner et al., 2021), suggesting that LDs may be involved in regulating sterol homeostasis under both biotic and abiotic stress conditions. The detection of sterol metabolism-associated proteins in LD proteomes is consistent with the ability of LDs to store SEs, thereby contributing to the regulation of sterol homeostasis in plant cells.

Proteins involved in the biosynthesis of antimicrobial compounds were also identified in leaf LD proteomes. Among them, the α-dioxygenase 1 (α-DOX1) and caleosin 3 (CLO3), which cooperatively synthesize the phytoalexin 2-hydroxy-octadecatrienoic acid (2-HOT), were shown to localize to LDs during infection with *C. higginsianum* and *P. syringae* in *Arabidopsis* (Shimada et al., 2014; Fernández-Santos et al., 2020). They are also enriched in LD fractions in the *tgd1–1 sdp1–4* mutant in response to *P. syringae* or *B. cinerea* infections (Scholz et al., 2025). Similarly, the protein phytoalexin deficient 3 (PAD3), involved in the last step of camalexin biosynthesis (Schuhegger et al., 2006), co-localizes with LDs upon *P. syringae* infection (Fernández-Santos et al., 2020). Collectively, these findings indicate that although key enzymes of lipid-derived defense pathways are associated with LDs under various conditions, including abiotic stresses (**Figure 2**), their selective enrichment and re-localization upon pathogen infection point to a pathogen stress-specific remodeling of the LD proteome, potentially linked to the activation of antimicrobial metabolism.

PUX10, the UBX domain-containing protein 10, which is involved in proteasome-dependent degradation of oleosins during seed germination (Deruyffelaere et al., 2018; Kretzschmar et al., 2018), was identified in leaf LD fractions isolated from *P. syringae*-infected and senescent *Arabidopsis* leaves (Fernández-Santos et al., 2020). However, this proteomic analysis is not quantitative and does not address whether PUX10 abundance changes in response to these conditions. Moreover, PUX10 recruits CDC48 to LDs, where CDC48 mediates the extraction of ubiquitinated oleosins for proteasomal degradation; Oleosins are largely absent from leaves LD proteomes described in **Figure 2**, where caleosins and LDAPs instead fulfill the structural role played by oleosins in seeds (Horn et al., 2013; Gidda et al., 2016; Brocard et al., 2017; Fernández-Santos et al., 2020). Whether the PUX10–CDC48 pathway similarly mediates the degradation of caleosins and LDAPs, or of other LD proteins during biotic and abiotic stress conditions, remains an open question.

Myosin-binding proteins were also identified in several LD proteomes. MYOB14 was detected in the LD proteome of the *hise1* mutant under standard growth conditions, and its localization to LDs was confirmed by confocal microscopy. LD localization was further validated for additional MYOB family members, including MYOB1, MYOB2, MYOB3, and MYOB5 (Omata et al., 2024). Interestingly, MYOB14 was also previously identified in LD proteomes associated with *P. syringae* infection and senescence (Fernández-Santos et al., 2020). The recurrent identification of MYOB proteins in distinct LD proteomes suggests that their association with LDs is not restricted to a specific stress condition and may reflect a broader functional link between LDs and the actomyosin trafficking machinery.

Finally, a subset of stress-responsive but poorly characterized proteins was found to associate with LDs. These include cytochrome B5 isoform E (CB5-E), early responsive to dehydration 7 (ERD7), LD-localized NTF2 family protein (LDNP), lipid droplet and stomata 1 (LDS1), lipid droplet-associated lipase 2 (LIDL2), the LD-associated methyltransferases LIME1 and LIME2, as well as unsaturated fatty-acids methylase/desaturase 1 (UFAMD1) and unsaturated fatty-acids oxygenase 1 (UFAO1) (**Figure 2**). Among these proteins, CB5-E, LDNP, LIDL2, UFAO1 and UFAMD1 were exclusively detected in the LD proteome of mutants with high LD levels (Omata et al., 2024; Scholz et al., 2025). In contrast, LIME1/2 and ERD7 were also identified in LD fractions from wild-type plants under drought stress and senescence, respectively (Brocard et al., 2017; Doner et al., 2021). Upon *B. cinerea* infection, the *tgd1–1 sdp1–4* mutant displays LDs enriched in CB5-E, ERD7, LDNP, and LIME1/2 proteins (Scholz et al., 2025), whereas infection with *P. syringae* leads to the accumulation of LDs enriched in CB5-E, ERD7, and LDS1 (Fernández-Santos et al., 2020; Scholz et al., 2025). The role of these proteins in pathogen infection remains to be determined.

Overall, the limited number of proteomic studies currently available does not allow the identification of a clear pathogen-specific LD protein signature except for proteins involved in phytoalexin metabolism that appear to be consistently enriched upon pathogen infection. Studies performed in high-oil-content backgrounds nevertheless allow valuable quantitative comparisons between unstressed and stressed conditions, although differences observed in LD proteomes from these mutants may also reflect the altered lipid homeostasis associated with these genetic backgrounds.

#### 2.1.3 Pathogen-induced transcriptional regulation of lipid droplet-associated genes

**Figure 3:**
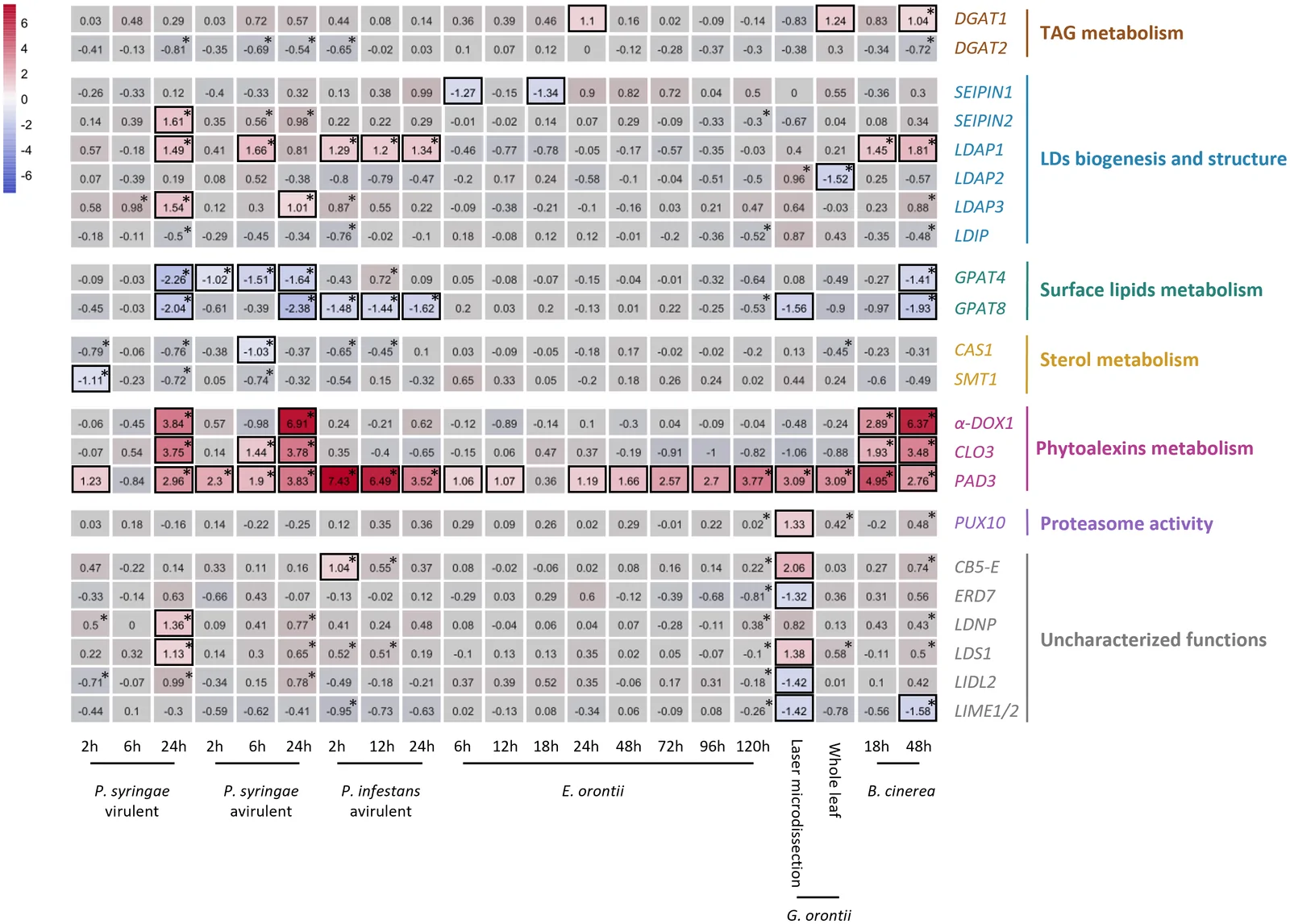
Transcriptional regulation of LD-associated genes during pathogen infection. Heatmap showing mean Log2 fold-change values of gene expression between infected and control conditions for genes associated with LDs. Normalized data were extracted from AtGenExpress Plant-Pathogen Interaction compendium using the BAR Toronto eFP Browser. Data available in this database were obtained using the Affymetrix’s GeneChip microarray platform and normalized using the standard Affymetrix MAS5.0 algorithm with a target value of 100 (Toufighi et al., 2005; Winter et al., 2007). In this single heatmap, we compile five independent experiments performed on *Arabidopsis thaliana* leaves infected with different pathogens, each compared to its own mock-inoculated control: *Pseudomonas syringae* pv. *tomato* DC3000 (virulent strain) and *P. syringae* pv. *tomato avrRpm1* (avirulent strain) (n = 3 plants per condition); *Phytophthora infestans* (n = 3); *Erysiphe orontii* (powdery mildew) (n = 3); *Golovinomyces orontii* (powdery mildew) (n = 2). Whole infected leaves were sampled in those experiments, excepted for (Chandran et al., 2010), where epidermal cells containing haustoria were also laser-microdissected - and collected at 5 days post-inoculation (dpi); *Botrytis cinerea* (n = 3). Time points indicate hours post-inoculation (h). Values correspond to expression fold changes relative to mock-inoculated controls. The color scale represents mean Log2 fold-change values, ranging from +6 (red, upregulated) to −6 (blue, downregulated). Statistical significance of expression changes was assessed independently within each experiment using moderated t-tests with empirical Bayes variance shrinkage (limma package, Bioconductor). Genes with an FDR-adjusted p-value < 0.05 are marked with an asterisk (*). In addition, to highlight the major changes, only values with an absolute Log2 fold-change ≥ 1 are shown in color; values below this threshold are shown in grey, regardless of statistical significance. Given the limited number of replicates in some experiments (n = 2 for *G. orontii*), statistical power remains constrained for these comparisons.

While integrating proteomic and transcriptomic data would provide a more complete understanding of LD dynamics upon pathogen infection, the lack of studies combining both approaches makes it difficult to establish any direct comparison. Nevertheless, the expression of genes encoding LD-associated proteins identified in proteomic studies can be explored in publicly available data and offers valuable insights into the regulation of LD dynamics during pathogen infection. Previous studies exploring publicly available microarray data have highlighted the enhanced transcription of stress-responsive genes functionally associated with LDs such as *CLO3* and *α-DOX1* during *B. cinerea* infection (Sham et al., 2014). Yet, the transcriptional regulation of other LD-related genes has not been described. We thus examined the expression patterns of these genes under biotic stress conditions using transcriptomic datasets from the AtGenExpress Plant-Pathogen Interaction compendium (Toufighi et al., 2005; Winter et al., 2007) and visualized them as a heatmap (**Figure 3**). This dataset covers the *Arabidopsis* leaf transcriptome at multiple time points post-infection by diverse pathogens: the bacteria *P. syringae*, the oomycete *P. infestans*, the biotrophic fungi *Erysiphe orontii* and *G. orontii* (Chandran et al., 2010), as well as the necrotrophic fungus *B. cinerea*. We focused on genes involved in TAG biosynthesis (*DGAT1* and *DGAT2*), in LD biogenesis (*SEIPINs*, *LDAPs* and *LDIP*), as well as genes encoding proteins previously detected in leaf LD proteomes (**Figure 2**).

Among LD biogenesis-related genes, the expression of *LDAP1* is consistently and significantly increased in response to infection by diverse pathogens including *P. syringae*, *P. infestans* and *B. cinerea*. *LDAP3* and *SEIPIN2* also display a transcriptional response, particularly during later stages of *P. syringae* infection. *DGAT1* expression is also significantly upregulated in response to *B. cinerea* infection. The up-regulation at the transcriptional level of genes involved in TAG biosynthesis and LDs biogenesis across different pathosystems is consistent with a broad accumulation of TAGs observed in response to diverse pathogens, as described above.

An even stronger transcriptional response is observed for genes involved in phytoalexin biosynthesis. Among these, *PAD3* stands out as a well-established stress marker, consistently and strongly induced during pathogen attack. Indeed, its upregulation is a hallmark of defense responses against a broad spectrum of pathogens (Zhou et al., 1999; Schuhegger et al., 2006). Microarray data from the AtGenExpress project confirm this pattern, with *PAD3* displaying robust and sustained induction across all pathogen treatments (**Figure 3**). Interestingly, *CLO3* and *α-DOX1* also exhibit coordinated patterns of transcriptional induction across some, but not all, pathogens. Indeed, their expression is specifically and strongly enhanced in response to *B. cinerea* and *P. syringae*, whereas no clear transcriptional induction is observed during *P. infestans*, *E. orontii* and *G. orontii* infections. The differential regulation of *CLO3* and *α-DOX1*, compared to the broad induction of *PAD3*, may highlight diverse infection strategies employed by pathogens.

Some LD-associated genes with uncharacterized functions, such as *CB5-E*, *LDS1*, and *LDNP*, also appear to be transcriptionally induced during *P. syringae* or *P. infestans* infections. Further validation would be needed to confirm the biological relevance of these changes.

In contrast, *GPAT4* and *GPAT8*, involved in surface lipid metabolism display significantly downregulated expression upon various pathogen infections. Given that loss of GPAT4/GPAT8 function compromises cuticle integrity and increases susceptibility to several pathogens (Li et al., 2007), it could represent a pathogen-induced weakening of the cuticular barrier. Further studies are needed to clarify the functional significance of this transcriptional response.

Other LD-proteome related genes do not show strong or consistent transcriptional responses across pathogens. Collectively, these data indicate that LD-associated genes are selectively regulated in response to pathogen infection: defense-related metabolic enzymes-encoding genes are strongly induced, genes encoding core components of TAG synthesis and LD structure are only moderately upregulated at the transcriptional level, whereas genes encoding LD-located proteins with other functions do not show significant transcriptional responses. This suggests that LD accumulation and proteome remodeling under pathogen stress may depend not only on transcriptional regulation, but also on other mechanisms including translation regulation, proteasomal degradation, or protein subcellular relocalization of LD proteins upon infection.

Together, these observations indicate that pathogen-induced TAG accumulation and LD proliferation represent a major metabolic adjustment during plant-pathogen interactions. However, whether this extensive LD remodeling primarily benefits the host by supporting defense and cellular homeostasis or, instead, provides exploitable metabolic resources for invading pathogens, remains an open and central question. Although only a limited number of studies have directly addressed the functional roles of LDs in plants during pathogen infection, accumulating evidence suggests that they play both roles depending on the pathogen, as described below.

### 2.2 LDs are hubs of plant defense or pathogen invasion during bacteria and fungi infection

While the roles of LDs in mammalian (particularly human) immunity are well characterized (Pereira-Dutra et al., 2019; Tan et al., 2024), plant LDs have only recently been recognized as active participants in defense responses, notably during bacterial and fungal infections (Shimada and Hara-Nishimura, 2015; Scholz et al., 2025). Xue et al. (2025) provided evidence for an association between LD accumulation and plant defense during *Golovinomyces orontii* infection. Spore production was significantly increased in the *dgat1* mutant, which is impaired in TAG synthesis and LD biogenesis, indicating that LDs can contribute to the restriction of pathogen growth and host colonization. However, the mechanisms underlying this protective effect remain unexplored. Nevertheless, the growing characterization of the plant LD proteome, together with current knowledge of lipid-mediated immunity, suggests several possible mechanisms by which LDs could be involved in plant-pathogen interactions. These include the provision of lipid precursors for membrane remodeling during defense responses, the regulation of lipid-derived signaling pathways, and the recruitment of immune-related proteins to LD surfaces, contributing to the activation and coordination of plant immune responses (Kuźniak and Gajewska, 2024).

#### 2.2.1 LDs may be cytoprotective players during infection in plants

LDs mediate cytoprotective functions by buffering toxic free FAs in animals (Olzmann and Carvalho, 2019) and by contributing to protein quality control, notably through the sequestration and clearance of misfolded or damaged proteins, as shown in yeast (Geltinger et al., 2020; Garcia et al., 2021), metazoan and human cells (Roberts and Olzmann, 2020). This cytoprotective role of LDs in sequestering free FAs has also been demonstrated in plants, albeit in different contexts, such as nitrogen deprivation (Coulon et al., 2024) or growth (Fan et al., 2013). LDs may fulfill a similar function during pathogen infection by sequestering free FAs, especially polyunsaturated ones, which can accumulate during oxidative bursts and cause membrane damage (Fan et al., 2014; Olzmann and Carvalho, 2019). These potentially toxic compounds are safely stored in the form of TAGs or SEs within LDs (Listenberger et al., 2003; Fan et al., 2014; Xu and Shanklin, 2016), although the ultimate fate of these sequestered fatty acids remains largely unsolved.

#### 2.2.2 LDs may participate in building or modifying physical barriers

The cuticle, cell wall, and plasma membrane represent the first physical barriers encountered by pathogens such as bacteria and fungi (Yeats and Rose, 2013; Ziv et al., 2018). The plant cuticle, composed primarily of cutin monomers and waxes derived from very-long-chain FAs, including alkanes, is synthesized by epidermal cells and deposited at the cell surface (Domínguez et al., 2011). Pathogen infection can induce LD accumulation and enhance cuticular deposition, as observed during *Colletotrichum acutatum* infection in *Citrus sinensis* petals (Marques et al., 2016). Genetic alterations that reduce cutin content or modify wax composition frequently lead to altered disease outcomes, increasing susceptibility to bacterial pathogens while conferring enhanced resistance to some necrotrophic fungi. For example, cutin-deficient *Arabidopsis* mutants, *att1*, *bdg*, and *lcr*, displayed enhanced susceptibility to *P. syringae* but increased resistance to *B. cinerea* (Tang et al., 2007). LDs, as reservoirs of fatty acids and harboring enzymes required for cutin biosynthesis (Fernández-Santos et al., 2020; Zhou et al., 2026), could be involved in the resistance to the bacteria. Indeed, the loss of LD-localized glycerol-3-phosphate acyltransferases *GPAT4* and *GPAT8*, which are required for cutin biosynthesis during *P. syringae* infection, compromises cuticle integrity and alters host resistance to the bacteria (Li et al., 2007; Fernández-Santos et al., 2020). LDs could also be involved in the modulation of wax composition, impacting defense outcomes. Indeed, overexpression of *AtCLO3* increases wax alkane content and enhances resistance to *Alternaria brassicicola*, whereas its silencing promotes fungal growth and alters bacterial susceptibility (Hanano et al., 2015, 2023). Together, these studies support a model in which lipid remodeling of the cuticle, possibly mediated by LDs (Zhou et al., 2026), represents a dynamic component of plant defense. The consequences of this remodeling, however, appear to be pathogen-dependent, as alterations in cuticle composition can either enhance resistance or increase susceptibility depending on the infection strategy of the invading pathogen.

Beyond their potential contribution to cuticle formation, LDs may also participate in the remodeling of cellular membranes, another essential component of plant physical barriers. During infection, cellular demand for membrane lipids increases sharply as a consequence of organelle remodeling and increased vesicle trafficking (Gu et al., 2017; Bhandari and Brandizzi, 2024). LDs could meet this demand by supplying FAs to synthesize phospholipids necessary for host membrane biogenesis and repair, thereby supporting membrane expansion associated with defense-related vesicle trafficking and the formation of specialized apoplastic structures, including papillae, pathogen-associated cell wall appositions, and extracellular vesicles (Bhandari and Brandizzi, 2024). Sterol esters stored in LDs can also be mobilized to cellular membranes (Yang and Benning, 2018; Olzmann and Carvalho, 2019), contributing to the regulation of membrane lipid composition and fluidity. Microscopy analyses indicate that LDs undergo dynamic relocalization upon oomycete infection and become enriched at the cell periphery, in close proximity to the plasma membrane-derived interfaces at infection sites (Pandey et al., 2021), suggesting that LDs could transport lipids or proteins to the plasma membrane. This is further supported by the fact that, according to proteomic analyses, proteins typically associated with the plasma membrane, including syntaxins, synaptotagmin 1, and hypersensitive-induced response proteins HIR1 and HIR2 have notably been shown to be enriched in LD fractions during infections by *P. syringae* (Fernández-Santos et al., 2020).

#### 2.2.3 LDs contribute to the production of antimicrobial products and modulate defense signaling

LDs have been proposed to act as metabolic hubs for the synthesis, storage, and mobilization of defense-related lipids, including oxylipins, and lipophilic antimicrobial compounds such as phytoalexins (Shimada et al., 2014; Fernández-Santos et al., 2020). As an example, in *Arabidopsis*, the phytoalexin camalexin biosynthesis induced in response to fungal and bacterial infections relies on LD-associated enzymes such as PAD3 (Fernández-Santos et al., 2020). *Pad3–1* mutants display enhanced susceptibility to several fungi, most notably *A. brassicicola* and *B. cinerea*, but not to the bacteria *P. syringae*, highlighting pathogen-specific contributions of camalexin metabolism (Zhou et al., 1999; Fernández-Santos et al., 2020).

LDs also host oxylipin-producing enzymes such as α-DOX1 and CLO3, which catalyze lipid oxidation directly at the LD surface (Shimada et al., 2014). These enzymes cooperate to produce oxylipins such as 2-HOT, which display antifungal activity and contribute to plant defense (Shimada et al., 2014; Hanano et al., 2015). Consistently, LDs enriched in α-DOX1 and CLO3 accumulate upon fungal infection in *Arabidopsis* (Partridge and Murphy, 2009; Sham et al., 2015) and upon bacterial infection in *Arabidopsis, N. benthamiana* and *N. tabacum* (De León et al., 2002; Hamberg et al., 2003; Fernández-Santos et al., 2020), highlighting their role as putative sites of oxylipin production during pathogen infection. In rice, the caleosin-like peroxygenase OsPXG9 is a LD-associated enzyme involved in oxylipin metabolism (Tran et al., 2023). It utilizes lipoxygenase-derived FA hydroperoxides to produce epoxy- and hydroxy-FAs, which have antifungal activity. In addition to this role in oxylipin biosynthesis, OsPXG9 contributes to the turnover of lipid peroxides (i.e., reactive fatty acid hydroperoxides derived from polyunsaturated fatty acids) and thereby participates in the control of cellular redox status. Notably, *OsPXG9* expression is downregulated in response to biotic signals such as flg22, and its expression is negatively correlated with jasmonate biosynthetic genes. This suggests that, although OsPXG9 generates defense-related oxylipins, its lipid peroxide-scavenging activity may dampen the oxidative burst required for effective immune responses. Together, these findings indicate that LD-associated peroxygenases can fine-tune plant immunity by balancing oxylipin production and redox homeostasis. Consistent with this concept, in cotton, the LD-associated protein GhLDAP3 physically interacts *in planta* with the Golgi vesicle-membrane-localized cytochrome GhB561-11, a redox-active component of the ascorbate antioxidant generation machinery (Liu et al., 2025). This interaction could functionally link LDs to cellular redox homeostasis. Simultaneous knock-down of *GhLDAP3* and *GhB561–11* impairs ascorbate regeneration, leading to reduced antioxidant capacity and sustained accumulation of H_2_O_2_, which in turn enhances resistance to *Verticillium dahliae*, highlighting the trade-off between redox buffering and defense response activation (Liu et al., 2025).

Additional defense-related proteins associate with LDs, including the calcium-dependent protein kinase AtCPK1, which localizes to both LDs and peroxisomes in different cellular contexts, including roots and suspension-cell cultures (Coca and San Segundo, 2010). *AtCPK1* expression is induced by fungal elicitors from *Fusarium oxysporum* and modulates susceptibility to fungal and bacterial pathogens, *F. oxysporum*, *B. cinerea*, and *P. syringae*, through SA-dependent signaling. Microscopy analyses revealed a close spatial association between LDs and peroxisomes, suggesting potential interactions between these organelles during immune responses, although the underlying mechanisms remain unclear, and effects on number and lipid accumulation remain to be quantified in this study (Coca and San Segundo, 2010).

#### 2.2.4 Beyond host defense: LDs as emerging resources for bacterial and fungal pathogens

Although LDs are often associated with defense-related functions, very few studies suggest that they may also exert pathogen-beneficial effects. Some LD-localized enzymes can promote susceptibility. Several GDSL (Glycine–Aspartate–Serine–Leucine)-type lipases associated with LDs have been shown to act as negative regulators of plant immunity. In rice, LD-localized lipases such as OsGLIP1 and OsGLIP2 (GDSL-like-motif lipase 1 and 2) negatively regulate defense against bacterial blight (*Xanthomonas oryzae*) and rice blast (*Magnaporthe oryzae*), potentially through interference with JA- or SA-dependent signaling pathways, as indicated by compromised expression of pathogen-related genes (Gao et al., 2017). Lipidomic analyses further showed that these lipases modulate the abundance of multiple lipid species, suggesting that their impact on immunity is mediated through alterations in lipid homeostasis. Similarly, the pepper GDSL lipase CaGLIP1 is induced during pathogen infection, and its silencing enhances resistance to *Xanthomonas campestris* pv. *vesicatoria* and to the biotrophic oomycete *Hyaloperonospora parasitica*, while its overexpression in *Arabidopsis* leads to increased susceptibility to both pathogens (Hong et al., 2008). Together, these findings support a model in which LD-associated lipases can promote pathogen susceptibility by reshaping lipid metabolism and interfering with defense signaling pathways. LDs, as reservoirs of energy-rich neutral lipids, could also potentially serve as metabolic resources for invading pathogens. In animal systems, some intracellular pathogens actively exploit host LDs to access FAs required for their growth and replication (Roingeard and Melo, 2017; Safi et al., 2023). In plants, direct evidence for pathogen-mediated exploitation of host LDs remains limited, but some studies suggest that fungal and oomycete pathogens can mobilize host lipid reserves: *Colletotrichum* species and *Botrytis cinerea* both rely on host-derived lipids to support the formation of infection structures such as appressoria (Kuźniak and Gajewska, 2024). Similarly, the oomycete *Phytophthora infestans* induces LD mobilization in potato guard cells, correlating with TAG breakdown and a reduction in LD size during sporangiophore emergence, suggesting that these pathogens exploit host LDs (Yang et al., 2021).

Collectively, available data mostly position LDs as dynamic hubs of plant immunity against bacterial and fungal pathogens. LDs integrate metabolic, structural, and signaling layers of defense. Their rapid relocalization to sites of pathogen entry, as described by Shimada et al. (2014), supports the view that LDs function as mobile immune organelles enabling localized and timely responses. However, the same properties that make LDs effective immune organelles i.e., mobility, lipid flux control, and enrichment in metabolic and signaling factors, also render them attractive targets for intracellular pathogens such as viruses, which frequently hijack host lipid hubs to support replication and assembly.

## 3 LDs and viral infection in plants: an emerging research frontier

### 3.1 LD involvement in endomembrane remodeling and viral replication compartment biogenesis?

Almost all positive-stranded RNA viruses [(+) RNA viruses] remodel host endomembranes to form viral replication compartments (VRCs), defined as dynamic membrane-rich structures that support viral replication and movement as shown for potyviruses (Xue et al., 2023). These structures create a favorable environment for viral replication by concentrating host and viral components while shielding replication intermediates from antiviral defenses such as RNA silencing. Interestingly, LDs have been shown to serve as VRC and/or virion assembly platforms for several animal viruses, notably *Flaviviridae* members such as hepatitis C, dengue or Zika viruses (Miyanari et al., 2007; Cloherty et al., 2020; Hsia et al., 2024). The assembly of hepatitis C viral particles begins at the surface of LDs, where multiple viral proteins co-localize. LDs serve as a primary source of TAGs incorporated into lipoviroparticles (Vieyres and Pietschmann, 2019). Furthermore, LDs appear to serve as storage sites for viral capsid proteins (Iglesias et al., 2015). Poliovirus (PV) recruits LDs to form VRCs, through membrane contact sites (MCSs) that enable transfer of FAs originating from the lipolysis of LD-stored TAGs to VRCs, where they are incorporated as polar lipids to VRC membranes (Laufman et al., 2019). LD recruitment is driven by poliovirus proteins that tether the VRCs to the LDs, and the inhibition of the MCS formation between LDs and VRCs impedes poliovirus replication (Laufman et al., 2019). Dengue virus promotes lipophagy to enhance its replication (Reviewed in Hsia et al., 2024). This process reduces LD size and lowers intracellular TAG levels, while simultaneously increasing the concentration of free FAs. These FAs are then degraded through β-oxidation to produce ATP, which likely supports efficient viral replication. Blocking autophagy hinders dengue virus replication, but this effect can be reversed by adding external free FAs, suggesting that the virus reroutes FAs generated by the selective autophagy of LDs (Cloherty et al., 2020).

In plants, depending on the virus, VRCs can originate from different organelles, including the ER, mitochondria, chloroplasts, peroxisomes, or vacuoles, and are associated with extensive membrane remodeling (Xu and Nagy, 2014). Emerging evidence indicates that plant viruses with different genome types manipulate LDs to support these processes, though whether they serve identical functions to animal systems remains an active area of investigation.

#### 3.1.1 Positive-sense single-stranded RNA viruses

Turnip mosaic virus (TuMV, family *Potyviridae*) provides a well-characterized example of plant viruses that utilize the host endomembrane system to produce replication vesicles involved in cell-to-cell movement (Grangeon et al., 2013). Recently, Jambou et al. (2026) showed an accumulation of LDs within TuMV-induced VRCs in both *A. thaliana* and *N. benthamiana*, accompanied by an increase in TAG levels during infection in *N. benthamiana*. TuMV infection assays in *Arabidopsis* mutants impaired in LD biogenesis further showed that LD-associated factors, such as LDAP1 and SEIPIN positively contribute to infection. Indeed, TuMV propagation is reduced in mutants with decreased LD abundance. Together, these findings provide the first evidence for a proviral role of LDs in potyvirus infection.

Membrane remodeling during VRC assembly requires tight control over lipid composition and membrane properties. ER shaping proteins such as the reticulon-like protein HVA22a and the ER fusogen RHD3 promote TuMV propagation by facilitating the maturation of viral vesicles (Movahed et al., 2019; Xue et al., 2024). Notably, RHD3 localizes to ER subdomains associated with LD biogenesis (Wright et al., 2025), and proteomic analyses have identified reticulon-like proteins (HVA22a) and RHD3 within LD-enriched fractions (Omata et al., 2024), suggesting LDs may supply key structural proteins to VRC assembly sites.

Lipid metabolism via lipins (phosphatidic acid phosphohydrolases, PAHs) further highlights this connection. These enzymes convert phosphatidic acid into diacylglycerol, a precursor of TAGs, and are involved in LD formation. In yeast, lipin deficiency leads to excessive ER membrane proliferation, which enhances tomato bushy stunt virus (TBSV, family *Tombusviridae*) replication by increasing membrane availability for VRC assembly (Chuang et al., 2014), while *AtPAH2* overexpression in *N. benthamiana*, reduces TBSV accumulation (Chuang et al., 2014). Mutations in *PAH* genes in plants disrupt LD formation and induce ER expansion (Fan et al., 2026). While these genetic links demonstrate an interplay between PAH activity and viral replication, it remains to be determined whether altered LD numbers directly impact VRC biogenesis or if this phenotype is an indirect consequence of ER membrane expansion.

Additionally, unsaturated FAs are essential for replication vesicle formation in brome mosaic virus (BMV, family *Bromoviridae*)-infected cells (Lee et al., 2001). Other (+)ssRNA viruses similarly alter LD distribution or host lipid profiles: melon necrotic spot virus (MNSV, family *Tombusviridae*) infection triggers the clustering of LDs with ER and mitochondria (Gómez-Aix et al., 2015), which we hypothesize may reflect a localized metabolic role coupled to supply energy or lipid precursors to replication complexes. Likewise, cowpea mosaic virus (CPMV, family *Secoviridae*) relies on continuous *de novo* lipid synthesis for ER proliferation and replication vesicle formation (Carette et al., 2000). Based on these observations, it is tempting to speculate that LDs serve as dynamic lipid reservoirs supporting this membrane expansion, though direct lipid flux between LDs and expanding VRCs remains to be demonstrated.

#### 3.1.2 Negative-sense single-stranded RNA viruses

Beyond (+)ssRNA viruses, negative-strand RNA viruses also target host LDs. Dai et al. (2024) demonstrated that the nucleocapsid (N) protein of Valsa mali negative-strand RNA virus 1 (VmNSRV1, family *Phenuiviridae*) directly binds to LDs in both fungal and plant cells, causing architectural rearrangement that result in fewer, larger LDs. We hypothesize that this N protein-driven reorganization alters local lipid composition or microenvironments to favor viral replication; however, the precise functional benefit of this LD remodeling awaits further experimental confirmation.

#### 3.1.3 Double-stranded RNA viruses

Plant double-stranded RNA viruses similarly could manipulate host LD proteins to stabilize lipid reserves crucial for infection. Rice black-streaked dwarf virus (RBSDV, family *Reoviridae*) induces significant accumulation of alpha-linolenic acid (C18:3) and linoleic acid (C18:2), both of which are required for viral replication and symptom development (Wang et al., 2024). Mechanistically, the RBSDV capsid protein P8 interacts with ZmLDAP2 to prevent its degradation via the ubiquitin-proteasome pathway. Silencing *ZmLDAP2* led to a ~70% reduction in viral accumulation (Wang et al., 2024). While these findings confirm that ZmLDAP2 stabilization promotes viral infection, whether this is a direct effect of ZmLDAP2 itself or an indirect consequence of altered LD biogenesis and fatty acid availability for VRC or virion assembly remains an open question.

### 3.2 LD involvement in potyvirus movement and PD targeting?

To invade the whole plant, plant viruses spread by moving between cells through PD, then entering sieve elements to travel with photoassimilates and reach sink tissues (Schoelz et al., 2011; Reagan and Burch-Smith, 2020).

Recent work by Jambou et al. (2026) underscores a pro-viral role of LDs in TuMV infection, particularly in cell-to-cell movement. Despite extensive investigations, whether potyviruses move between cells as virions or other movement complexes, such as ribonucleoproteins or 6K2 vesicles, remains unresolved, though evidence obtained so far indicates that viral replication and movement are closely interconnected (Reviewed in Xue et al., 2023). A survey of TuMV propagation in *Arabidopsis* mutants impaired in LD biogenesis revealed that two protein families crucial for LD biogenesis, LDAPs and SEIPINs, facilitate TuMV cell-to-cell movement (Jambou et al., 2026). Additional investigation is required to establish whether LDs directly participate in potyviral movement by transporting viral proteins or viral vesicles to PD, or act via indirect mechanisms.

The 6K2 protein of potyviruses is a transmembrane protein involved in ER rearrangement, leading to the generation of viral vesicles, important for both replication and intracellular and intercellular movement (Cotton et al., 2009; Grangeon et al., 2013; Wan et al., 2015). To exit the ER, 6K2 associates with COPII components, including Ras‐like small GTPase SAR1 and SEC24; these interactions are critical for maturation and movement of replication vesicles through PD (Jiang et al., 2015). Similarly, for the bymovirus wheat yellow mosaic virus (WYMV, family *Potyviridae*), the vesicle-forming protein P2 interacts with SAR1 to drive membrane rearrangements into replication vesicles and enable virus movement (Sun et al., 2014). Interestingly, both SAR1 and SEC24 are enriched in the LD proteome (Scholz et al., 2025), with SAR1 specifically enriched near the LD assembly sites at the ER (Wright et al., 2025). Based on these molecular links, we hypothesize that LDs could assist in trafficking viral movement proteins or replication vesicles within the *Potyviridae* family.

Other SNARE proteins, such as VTI11 and SEC22, interact with TuMV-6K2 and play essential roles in TuMV movement (Cabanillas et al., 2018). Importantly, SEC22 and VTI11 have been identified in *Arabidopsis* LD proteome together with other factors involved in potyvirus movement such as RTN3, RHD3, syntaxins, synaptotagmins, and cytoskeletal components. Most of these proteins facilitate vesicle docking or membrane reshaping at PD (Veerabagu et al., 2021; Omata et al., 2024). Remorin proteins have also been identified in LD-enriched fractions (Omata et al., 2024). Remorins constitute a large and functionally diverse protein family, however, specific members (particularly Group 1 remorins) accumulate in plasma membrane nanodomains in close proximity to PD, where they modulate the size exclusion limit (SEL) and regulate the cell-to-cell movement of potyviruses such as TuMV and Potato virus A (Rocher et al., 2022). Furthermore, it has been proposed that LDs might act in SEL regulation by delivering a subset of 1,3-β-glucanases (Paul et al., 2014; Veerabagu et al., 2021). Together, the presence of key PD-localized and trafficking-associated proteins within the LD proteome, combined with evidence that LDs dynamically accumulate near PD, raises several compelling questions such as: 1) Do LDs deliver host factors that modulate PD aperture? 2) Could they actively escort viral vesicles towards the PD?, 3) Do LDs themselves physically participate in intercellular virus movement? While these mechanisms remain speculative, they offer a plausible framework for how LDs may facilitate potyvirus spread.

As highlighted in a recent Commentary by May and Nagy (2026) of Jambou et al. (2026) paper, LD accumulation during plant virus infection may represent a contested host process, in which plants mobilize lipid remodeling as part of a biotic stress response while viruses redirect these organelles to create a membrane environment favorable for replication and movement.

In summary, several (+) RNA animal viruses have been shown to exploit LDs as lipid or energy sources for VRCs biogenesis or as physical scaffolds for virion assembly. We are only beginning to appreciate the similar involvement of LDs in plant virus infections. In plants, LDs cluster near viral vesicles and in VRCs, where they are hypothesized to act as metabolic hubs providing lipids or energy for synthesis and functioning of VRCs, structural scaffolds for VRC/virion assembly, or as cargo-trafficking platforms for viral proteins and replication vesicles. Although many of these mechanistic roles remain to be experimentally validated, validating LD-virus interactions opens exciting new avenues in plant pathology and cell biology.

## 4 Conclusion

Over the past decade, LDs have emerged as highly dynamic organelles whose functions extend far beyond simple lipid storage. Accumulating evidence from diverse plant-pathogen systems indicates that LDs undergo extensive remodeling during infection, accompanied by shifts in neutral lipid metabolism, changes in LD abundance, and selective recruitment of structural and defense-associated proteins. These observations collectively highlight LDs as central nodes connecting lipid metabolism with cellular stress responses.

**Figure 4:**
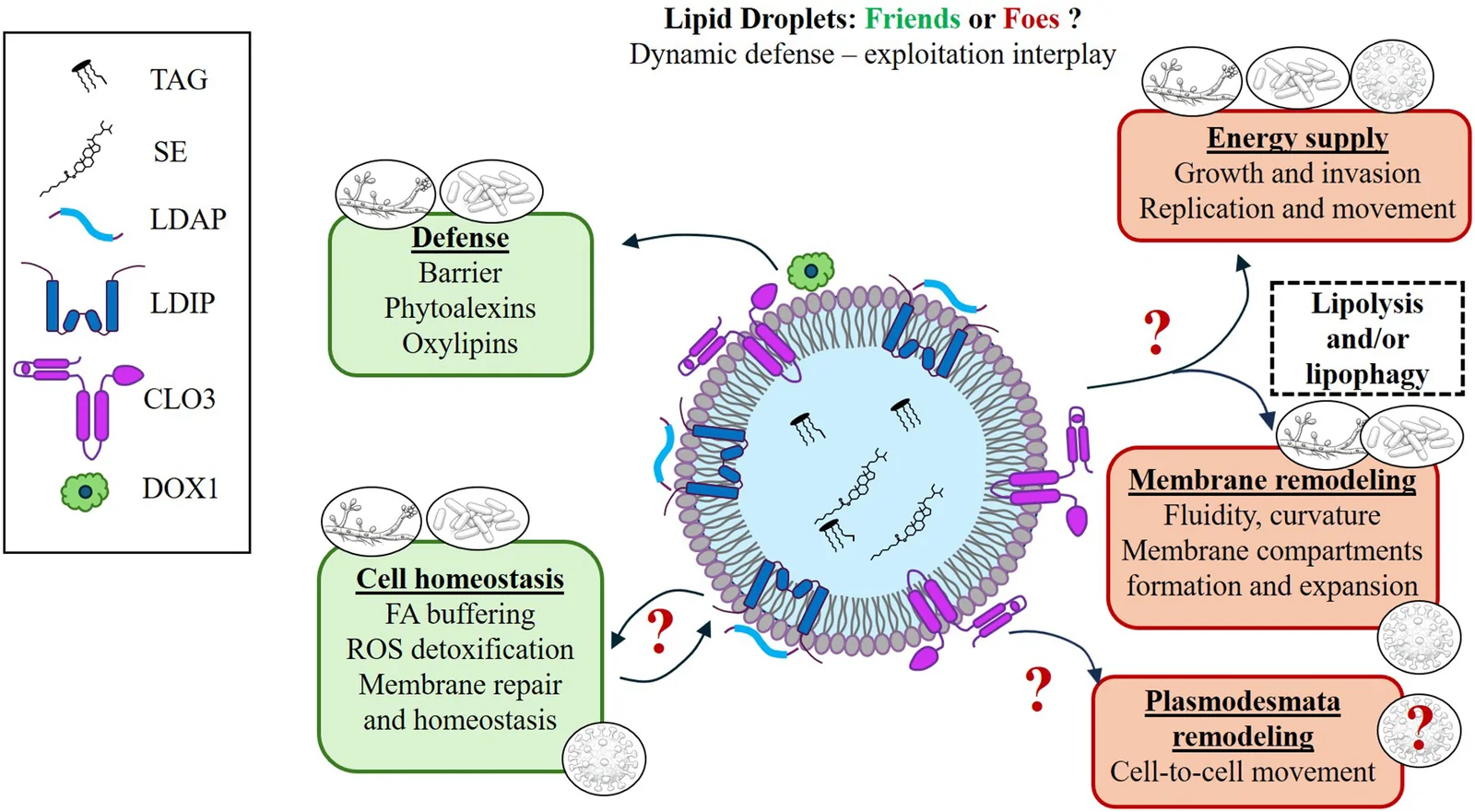
Potential involvement of LDs in plant defense and pathogen exploitation. LDs emerge as multifunctional organelles playing contrasting roles during plant-pathogen interactions. On the host side, LDs contribute to multiple layers of plant defense by supporting physical barrier formation, phytoalexin and oxylipin biosynthesis, redox homeostasis, FA buffering, membrane repair, and cellular homeostasis. Pathogen infection is frequently associated with extensive LD remodeling, including changes in abundance, lipid composition, intracellular distribution, and protein cargo. Conversely, the same properties that make LDs effective defense organelles may also render them valuable resources for pathogens. Bacterial, fungal, oomycete, and viral pathogens can potentially exploit LD-associated lipids, proteins, and metabolic activities to support growth, invasion, membrane remodeling, and infection. Emerging evidence further suggests that plant viruses may hijack LDs to facilitate virus replication compartment (VRC) biogenesis, membrane expansion, lipid trafficking, and cell-to-cell movement through PD. While several of these mechanisms remain hypothetical, current data position LDs as central regulators of the dynamic balance between host immunity and pathogen exploitation. CLO3, caleosin 3; DOX1, dioxygenase 1; FA, fatty acid; LDAP, lipid droplet associated protein; LDIP, LDAP-interacting protein; ROS, reactive oxygen species; SE, sterol ester; TAG, triacylglycerol.

The multifunctional nature of LDs places them at a strategic interface between host defense and pathogen exploitation (**Figure 4**). LDs may contribute to structural barrier formation, antimicrobial compound synthesis, and redox homeostasis, thereby supporting multiple layers of plant immunity. Yet their lipid-rich composition and integration within organelle networks also make them potentially attractive targets for pathogens seeking metabolic resources or membrane components required for their own proliferation. Understanding these diverse and context-dependent functions will require integrative approaches combining lipidomics, advanced imaging, and genetic analyses to resolve LD dynamics and lipid fluxes during infection. Such efforts will be essential to determine when LDs function primarily as defensive metabolic hubs, when they are actively manipulated by pathogens, and when they act as context-dependent regulators whose contributions vary according to the pathogen, the host, and the stage of infection.

## 5 Future perspectives

Although considerable progress has been made in understanding LD dynamics during plant-pathogen interactions, many fundamental questions remain unresolved. A major challenge is to distinguish whether LD remodeling primarily represents a host defense strategy or a process actively manipulated by pathogens, or both, whose function varies with the host, the pathogen lifestyle, and the stage of infection. Addressing these questions will require integrating spatial, temporal, and metabolic information rather than relying solely on static observations of LD abundance or morphology.

One important priority will be to determine the origin and fate of lipids mobilized during infection. Stable isotope labeling combined with *in vivo* lipid flux analyses could reveal whether FA stored in LDs are redirected toward membrane biogenesis, β-oxidation, oxylipin production, or other defense-related metabolic pathways. Such approaches would provide direct evidence for the metabolic contribution of LDs during immune responses and pathogen colonization.

Future studies should also exploit recent advances in imaging technologies. High-resolution live-cell microscopy, super-resolution imaging, correlative light and electron microscopy (CLEM), and quantitative three-dimensional analyses will make it possible to monitor LD dynamics and characterize their interactions with the ER, peroxisomes, chloroplasts, mitochondria, PD, and pathogen-derived structures. These approaches should help determine how MCSs reorganize during infection and whether specific organelle interactions underlie distinct immune or susceptibility responses.

Another important question concerns whether microbial pathogens directly manipulate LD biology through the action of secreted effectors. While numerous bacterial, fungal, oomycete, and viral effectors have been shown to target host organelles and reprogram cellular metabolism, evidence for the direct targeting of LDs or LD-associated proteins remains remarkably limited. Identifying pathogen effectors that interact with LD structural proteins, lipases, or regulators of LD biogenesis and turnover will therefore represent an important avenue for future research. Combining effector interactomics, proximity labeling, quantitative proteomics, and live-cell imaging should help determine whether pathogens actively reprogram LD dynamics as part of their infection strategy or whether LD remodeling primarily reflects host metabolic responses.

Although this review focuses primarily on microbial pathogens, LDs may also participate in plant defense against herbivorous insects. JA, whose biosynthesis depends on peroxisomal β-oxidation of oxylipin precursors, is a central regulator of anti-herbivore defenses (Wang et al., 2019). As discussed above, LD-derived FAs may contribute indirectly to JA biosynthesis, suggesting that LD turnover could indirectly influence defense responses to insect feeding. Furthermore, caleosin/peroxygenases participate in oxylipin metabolism and have been implicated in wound responses as well as biotic stress responses, providing an additional link between LD biology and herbivore-induced defense signaling (Wasternack and Feussner, 2018; Hanano et al., 2023). However, direct evidence demonstrating that LD remodeling contributes to resistance against herbivorous insects remains scarce. Future studies should therefore determine whether insect attack induces specific LD remodeling and whether LD-associated proteins contribute directly to anti-herbivore immunity.

Finally, future work should move beyond descriptive analyses to identify the molecular mechanisms governing LD remodeling during infection. Combining forward and reverse genetics with quantitative lipidomics, proteomics, metabolomics, and interactomics will help identify regulators of LD biogenesis, degradation, and organelle communication. Comparative studies across bacterial, fungal, oomycete, and viral pathogens will further clarify which LD responses represent conserved components of plant immunity and which correspond to pathogen-specific adaptations. Particular attention should be paid to deciphering how pathogens reprogram LD metabolism to redirect lipid fluxes and organelle communication, as this emerging interface is likely to represent a key determinant of disease outcome and a promising target for the development of durable crop protection strategies.

## Glossary

2-HOT: 2-Hydroxyoctadecatrienoic acid
α-DOX1: α-Dioxygenase 1
ATP: Adenosine triphosphate
ATG8: Autophagy-related protein 8
BMV: Brome mosaic virus
CAS1: Cycloartenol synthase 1
CB5-E: Cytochrome b5 isoform E
CDC48: Cell Division Cycle 48
CLO1: Caleosin 1
CLO3: Caleosin 3
CPMV: Cowpea mosaic virus
DAG: Diacylglycerol
DGDG: Digalactosyldiacylglycerol
DGAT1: Diacylglycerol acyltransferase 1
DGAT2: Diacylglycerol acyltransferase 2
dsRNA: Double-stranded RNA
ER: Endoplasmic reticulum
ERD7: Early Responsive to Dehydration 7
FA: Fatty acid
GPAT4: Glycerol-3-phosphate acyltransferase 4
GPAT8: Glycerol-3-phosphate acyltransferase 8
HIR1: Hypersensitive-Induced Response protein 1
HIR2: Hypersensitive-Induced Response protein 2
HVA22a: Hordeum Vulgare Abscisic acid responsive protein 22a
JA: Jasmonic acid
LDAP: Lipid Droplet-Associated Protein
LD: Lipid droplet
LDIP: Lipid Droplet-Associated Protein-Interacting Protein
LDNP: Lipid Droplet-localized NTF2 family Protein
LDS1: Lipid Droplet and Stomata 1
LIDL2: Lipid Droplet-Associated Lipase 2
LIME1: Lipid droplet-associated methyltransferase 1
LIME2: Lipid droplet-associated methyltransferase 2
MAG: Monoacylglycerol
MCS: Membrane contact site
MGDG: Monogalactosyldiacylglycerol
MNSV: Melon necrotic spot virus
MYOB: Myosin-binding protein
OBL3: Oil Body Lipase 3
OLE: Oleosin
OsGLIP1: Oryza sativa GDSL-like lipase 1
OsGLIP2: Oryza sativa GDSL-like lipase 2
OsPXG9: Oryza sativa Peroxygenase 9
PAD3: Phytoalexin Deficient 3
PAH: Phosphatidic acid phosphohydrolase
PC: Phosphatidylcholine
PD: Plasmodesmata
PDAT1: Phospholipid: Diacylglycerol Acyltransferase 1
PM: Plasma membrane
PUX10: Plant UBX Domain-containing Protein 10
PV: Poliovirus
PXA1: Peroxisomal ABC transporter 1
RBSDV: Rice black-streaked dwarf virus
RHD3: Root Hair Defective 3
RNA: Ribonucleic acid
ROS: Reactive oxygen species
SA: Salicylic acid
SAR1: Secretion-Associated Ras-related GTPase 1
SDP1: Sugar-Dependent Protein 1
SE: Sterol ester
SEL: Size exclusion limit
SMT1: Sterol Methyltransferase 1
SNARE: Soluble N-ethylmaleimide-sensitive factor Attachment protein REceptor
TAG: Triacylglycerol
TBSV: Tomato bushy stunt virus
TuMV: Turnip mosaic virus
UFAO1: Unsaturated Fatty Acid Oxygenase 1
UFAMD1: Unsaturated Fatty Acid Methylase/Desaturase 1
VAP27: Vesicle-associated membrane protein-associated protein 27
VmNSRV1: Valsa mali negative-strand RNA virus 1
VRC: Viral replication compartment
WYMV: Wheat yellow mosaic virus
